# The role of the intestinal ultrasound in Crohn’s disease diagnosis and monitoring

**DOI:** 10.25122/jml-2021-0067

**Published:** 2021

**Authors:** Norbert Dacian Stenczel, Monica Roxana Purcarea, Laura Carina Tribus, Gabriela Hofer Oniga

**Affiliations:** 1. Department of Healthcare Marketing and Medical Technology, Carol Davila University of Medicine and Pharmacy, Bucharest, Romania; 2. Department of Nephrology, Carol Davila Clinical Nephrology Hospital, Bucharest, Romania; 3. Department of Gastroenterology, Bucharest Emergency University Hospital, Bucharest, Romania; 4. Department of Gastroenterology, Carol Davila University of Medicine and Pharmacy Bucharest, Romania

**Keywords:** Crohn’s disease, inflammatory bowel disease, abdominal ultrasound, intestinal ultrasound, CDAI – Crohn’s Disease Activity Index, HBI – Harvey Bradshaw Index, MRI – magnetic resonance imaging

## Abstract

Crohn’s disease is characterized by persistent or recurrent chronic inflammation that may affect any segment of the gastrointestinal tract. It has an oscillating evolution, with periods of activity alternating with periods of remission. Crohn’s disease has a significant impact on the economic status due to its increasing prevalence, often affecting young people. Suitable management for these patients involves frequent evaluations. Even though colonoscopy is the gold standard for the assessment of severity and mucosal healing, it is an invasive maneuver, not easily accepted by patients, and it does not have good repeatability. Intestinal ultrasound has the advantage of being non-irradiating, non-invasive, well-tolerated, cheap, and easy to repeat. Ultrasound parameters such as bowel wall thickness, intestinal wall architecture, intramural vascularisation, proliferation of mesenteric fatty conjunctive tissue, and intraperitoneal fluid can provide good information regarding the severity of the disease, the differentiation between remission and relapse, and its complications. Some of the latest studies show good correlations between ultrasound parameters and inflammation markers (C-reactive protein, fecal calprotectin) and clinical severity scores of Crohn’s disease. Consequently, the importance of intestinal ultrasound has increased lately, and recent studies support its use to evaluate the severity of inflammation, differentiate between active disease and relapse, monitor therapy response and guide treatment, evaluate prognosis, and diagnose complications.

## INTRODUCTION

Inflammatory bowel diseases are chronic immune-mediated inflammatory disorders of the gastrointestinal tract, characterized by an oscillating evolution, with periods of activity alternating with periods of remission. Their etiopathogenesis is unknown, the pathogenic immune substrate interfering with the microbiome and the environmental factors in people with a genetic predisposition. Inflammatory bowel diseases include two entities: Crohn’s disease and ulcerative colitis.

Crohn’s disease is characterized by a persistent or recurrent chronic inflammation, diffuse or occasionally granulomatous, that may affect any segment of the gastrointestinal tract, mainly localized at the level of the terminal ileum. The inflammation is divided into segments, alternating with healthy asymmetrical and transmural mucous areas (the classical “paving stone” aspect). Complications such as stenosis, abscesses, or intestinal or perianal fistulas may occur during evolution. Histologically, it is characterized by a discontinuous, focal, transmural distribution of chronic inflammation, sarcoid granuloma, cryptitis, and cryptic abscesses. The positive diagnosis is reached by gathering the clinical, laboratory data, the colonoscopy, and histologic aspects.

Inflammatory bowel diseases strongly impact the economic and social status since they generally affect young people in industrialized areas and are growing in incidence and prevalence [1].

Although the colonoscopy is the gold standard investigation for monitoring Crohn’s disease evolution, this is an invasive maneuver, more difficult to tolerate by patients both because of the associated risks and because of the preparation; therefore, it cannot be done many times. Other limitations of the endoscopic investigations are the inability to assess the impairment degree of the intestinal wall, the small intestine, and the complications at this level (in case of failure to intubate a stenotic ileum) [2]. Most such patients are young and must be re-examined frequently. Consequently, an additional method for diagnosis is necessary to monitor such people and that is safe, non-invasive, and easily tolerated. At the moment, there is no gold standard imagistic intervention for diagnosis and monitoring. However, the intestinal ultrasound provides clear information on the bowel impairment and extra-intestinal manifestations [3–5] and has the advantage of being non-irradiating, non-invasive, well-tolerated, cheap and easy to repeat.

## MATERIAL AND METHODS

We systematically searched the main databases (Google Scholar, PubMed, EMBASE) for studies assessing the diagnostic accuracy of intestinal ultrasound in Crohn’s disease. Electronic databases were evaluated from inception to December 2020 using keywords such as intestinal ultrasound, Crohn, inflammatory, and bowel diseases.

## RESULTS

### Intestinal ultrasound

The abdominal ultrasound is helpful in diagnosing an important variety of gastrointestinal disorders. Lately, it has become more and more essential in the diagnosis and monitoring of inflammatory bowel diseases. The ultrasound examination of the bowel may be done both by a probe with a convex orientation and 3–5 MHz frequency and a probe of 5–10 MHZ with linear orientation for the assessment of the five layers of the intestinal wall [6]. Almost all the segments of the bowel can be seen using the ultrasound evaluation, except for the jejunum and for the rectum that may be difficult to examine because of the adjacent structures.

Before the examination of the bowel with high-frequency probes, standard abdominal ultrasound with a 3.5–5 MHz probe is recommended. The standard abdominal ultrasound must be preprandial, in the morning or at least 6 hours postprandial, when the peristaltic is minimum, and the intraluminal air quantity is the lowest [7]. Gradual compression with the examination probe is recommended to reduce the air quantity in the bowel. It was proven that the administration of the intraluminal liquid or the use of the enteral contrast improves the demarcation of layers for the evaluation of the intestinal wall architecture and the detection of jejunal and colonic lesions in patients with inflammatory bowel diseases. However, such techniques are not yet being used in the current practice [8].

High-frequency abdominal ultrasound does not provide a continuous and complete assessment of the bowel. The ileocecal region and the sigmoid colon are visible in all patients. The ascendant and descendent colon may be seen in most subjects. Nevertheless, it is more difficult to identify the flexures (especially the splenic) because of the fixed position at the diaphragm by ligaments. The transverse colon may be observable in most patients, but a complete assessment is hard to get because of the variable anatomy. Rectal and anal regions cannot be assessed correctly from the ultrasound point of view because of the pelvic localization [9–10].

The five layers of the intestinal wall are shown in Table 1.

**Table 1:** Ultrasound imaging of the intestinal wall.

Layer echogenicity	Anatomic structure
**Hypoechogenic (liquid) or hyperecogenic (air)**	Lumen
**Hyperechogenic access**	Lumen-mucous transition
**Hypoechogenic**	Mucous
**Hyperechogenic**	Sub-mucous
**Hypoechogenic**	Musculara propria
**Hyperechogenic**	Muscularis propria/serous transition, adjacent structures (fat, peritoneum)

The small intestine and the colon may be differentiated by the colon haustra and Kerckring folds, which are specific to the small intestine. The measurement of the intestinal wall thickness must be done for inflammatory bowel diseases. The gaps of the measurements result from the compression degree the examiner performs using the probe and the technical details related to the ultrasound and equipment frequency [11]. When using the high-frequency probe and the linear orientation, the normal thickness of the intestinal wall is <3 mm, with a smaller diameter at the level of the jejunum, ileum, and proximal colon and bigger at the level of the sigmoid colon [12]. The physiological contractions in the bowel’s normal peristaltic that result in the thickening of the intestinal wall must be considered; this may lead to the wrong interpretation. Besides the diameter of the intestinal wall, its architecture and the architecture of the adjacent structures must also be assessed [13].

Several ultrasound parameters characteristic of inflammatory bowel diseases were discovered in time. The most used is the diameter of the intestinal wall, which is high and correlates well with the clinical activity scores of Crohn’s diseases such as the Harvey Bradshaw Index (HBI) and Crohn’s Disease Activity Index (CDAI) [14–16]. Especially for Crohn’s disease, the transmural inflammation leads to intestinal wall thickening both in its active forms and in the chronic or silent forms, being one of the best ultrasound parameters for assessing the inflammatory activity and disease prognostic [2]. Although the thickness of the intestinal wall is the most known parameter of bowel inflammation, there are no standardized measurements yet. This explains the high variation of the results depending on the examiner and is not surprising, considering that there is no international agreement regarding the place of the measurements and if they should be cross-section or longitudinal.

Another parameter is the architecture of the intestinal wall. The disorganization of the transmural architecture may be noticed in the case of Crohn’s disease. Modification of the intestinal wall layering is a sign of active inflammation. In this case, there is no standardization of alterations, determining a high variability of results for every study [12, 17]. Also, the loss of the normal stratification of the intestinal wall may be associated with the lack of compressibility by the transducer.

The inflamed intestinal wall, as for Crohn’s disease, has the ability to develop new blood vessels by angiogenesis, the activity of the disease correlating with the increased blood supply at this level [18, 19]. Therefore, the increased intramural blood supply is another ultrasound parameter of inflammatory bowel disease and may be evaluated with a Doppler ultrasound. This is a sign of active inflammation and correlates well with endoscopic, histologic modifications, and the CDAI score [20]. Quantification of the blood supply degree may be difficult, considering that it depends on several factors, including food ingestion. Nevertheless, the Limberg score provides a semi-quantitative characterization of the bowel blood supply, classifying the bowel blood supply in 4 different categories [6].

The assessment of the adjacent structures is extremely important. Modifications at the level of mesenteric fatty tissue may be noticed in inflammatory bowel diseases. Most frequently, we can notice the proliferation of the mesenteric fatty conjunctive tissue around the affected intestinal ansae, characteristic of active Crohn’s disease. Such modifications emerge during the activity periods of Crohn’s disease and disappear totally or partially during remission periods. The proliferation of the fatty conjunctive tissue appears as a hyperechogenic area surrounding the inflamed intestinal wall. Although the modifications at the level of the mesenteric extramural areas may be easily detected within the abdominal ultrasound, there are no standard characteristics to differentiate the severity degrees of the disease, leading again to a high variability depending on the examiner [17]. Transmural inflammation may determine the occurrence of the intraperitoneal liquid.

The mesenteric lymphatic ganglia that may be seen during the ultrasound is specific to the acute inflammation and also to the chronic inflammation of the intestine. They are observable as hyperechogenic round or oval structures, but they also occur in case of inflamma-tory bowel diseases in remission; this is the reason for which this is not considered to be a good parameter for monitoring. Nevertheless, for active Crohn’s disease, the lymphatic ganglia may have bigger diameters and may be hemorrhagic, sometimes sensitive to the pressure of the transducer [21].

### Intestinal ultrasound in Crohn’s disease

It was proven that intestinal ultrasound shows increased sensitivity and specificity both in the primary diagnosis of Crohn’s disease and the detection of complications such as stenosis, fistulas, and abscesses [22–26]. The most important marker for inflammation is the thickness of the intestinal wall [27]. Several studies showed increased sensitivity and specificity of the intestinal wall thickness regarding the diagnosis of Crohn’s disease (75–93% and 75–100%, respectively) using the colonoscopy modifications for comparison [28, 29]. A recent study that included 249 patients suspected of Crohn’s disease used colonoscopy and magnetic resonance imaging (MRI) as comparative investigations and showed an increased diagnosis value of the intestinal ultrasound for the detection of Crohn’s disease, with a 94% sensitivity, a 97% specificity, a positive predictive value of 97% and a negative predictive value of 94% [30].

The global sensitivity was studied in several meta-analyses, one of them with the range under the ROC curve of 0.94, indicating good diagnostic accuracy. In the most recent meta-analyses, the intestinal ultrasound showed 79.7% sensitivity and 96.7% specificity in diagnosing the patients suspected of Crohn’s disease and 89% sensitivity and 94.3% specificity within the initial management of diagnosed Crohn’s disease patients. In the study of Calabrese *et al*., the intestinal ultrasound identified ileal Crohn’s disease with a sensitivity and specificity of 92.7% and 88.2%, respectively, and colonic Crohn’s disease with a sensitivity and specificity of 81.8% 95.3%, respectively, thus with lower accuracy in the detection of proximal lesions [25].

These studies show that the intestinal ultrasound is an essential method for diagnosing Crohn’s disease in the primary assessment of patients with intestinal symptoms suggestive of this disorder. Moreover, with a higher negative predictive value, Crohn’s disease diagnosis may be excluded in patients with non-specific gastrointestinal symptoms and biomarkers such as C-reactive protein (CRP) and fecal calprotectin with normal values.

Regarding the appreciation of the activity and the severity of Crohn’s disease, the role of the intestinal ultrasound is controversial, possibly because it is not yet clear which is the best ultrasound method to assess the activity of the disease. Most ultrasound scores used at the moment cannot correctly indicate the severity of this disorder [31]. The thickness of the intestinal wall showed significant correlations from the statistical point of view with the CDAI score in children and young adults [32]. However, the thickness level of the intestinal wall and the length of the intestine visibly enlarged in the ultrasound as markers of the activity degree of the Crohn’s disease proved to be significantly correlated, but too weak with the clinic and biologic parameters within the assessment of the disease severity [33]. Several studies showed that the high blood supply of the enlarged wall assessed by Doppler ultrasound might be a qualitative method to appreciate the clinic activity of Crohn’s disease. Therefore, the thickness of the intestinal wall together with the vascular pattern may be useful for detecting the endoscopic activity of the disease [34]. Other studies that assessed the superior mesenteric artery flow as a semi-quantitative parameter confirmed the previous observations regarding the correlation between the severity of the disease and Doppler parameters [35, 36].

### Complication of Crohn’s disease

Extramural complications of Crohn’s disease, as well as abscesses and fistulas, may be easily identified during the intestine ultrasound examination.

*• Abscesses* are irregular, aperistaltic, and hypoechogenic regions with no blood supply that may contain visible ultrasound air (hyper-echogenic lines). The sensitivity of the ultrasound detection of abscesses in Crohn’s disease varies between 80 and 100%, and the specificity between 92 and 94% [23, 37], with a global sensitivity and specificity of 84% and 93%, respectively [38]. Moreover, it was proved that sensitivity is higher for detecting superficial intraperitoneal abscesses than intrapelvic or retroperitoneal profound abscesses; these are less visible because of the presence and overlapping of the air in the gastrointestinal tract [37, 39];
*• Fistulas* are characterized by hypoechogenic tracks between the intestinal ansae or originate at the intestinal level and are connected with other tissues or organs, such as the skin, the gall bladder, or the vagina. Moreover, they may contain air, detectable as hyperechogenic areas during ultrasounds. The detection of fistulous tracks during ultrasound depends on where they are situated, with a 67–82% sensitivity and a 90–100% specificity [23, 24]. A recent meta-analysis established a global sensitivity of 74% and specificity of 95% for the diagnosis of fistulas in Crohn’s disease [38];
- Intestinal ultrasound is also useful to identify *stenosis* both at the level of the small intestine and the colon. Intestinal stenosis may be caused by inflammation, fibrosis, or a mixture of the two. Within several studies that used surgical intervention for comparison, the sensitivity of the ultrasound diagnosis of the stenosis ranked between 75 and 100%, and the specificity between 89 and 91%, with a global sensitivity of 79% and specificity of 92% [23, 24]. The use of contrast-enhanced intestinal ultrasound increased the sensitivity of stenosis detection and their number. Therefore, the intestinal ultrasound with oral contrast agents diagnosed at least a stenosis in more than 10% of the patients and at least 2 stenoses in more than 20% of patients as compared to the intestinal ultrasound with no contrast agent, with a sensitivity of nearly 90% for the detection of only one stenosis and more than 75% for multiple stenoses [40, 41]. In a recent study, the sensitivity and specificity of contrast-enhanced ultrasound were 92.3% and 92.1%, respectively [25].

## DISCUSSION

### Monitoring the activity of Crohn’s disease

The existing data prove that intestinal ultrasound may play an important role in monitoring Crohn’s disease and assessing the treatment response. There are few studies in this sense, but the results look promising. During such a trial, the anti-TNF alpha biologic therapy was related to a significant reduction of the intestinal wall diameter size and the blood supply at this level. The ultrasound parameters were improved in 50% of the analyzed patients [42]. The treatment of the acute outbreak of the disease determined an improvement of the ultrasound parameters, namely the thickness of the intestinal wall, the blood supply, and the fibro-connective proliferation in patients who also showed a significant decrease of the HBI score in 3 months since the initiation of the therapy. Improvement of the ultrasound aspect also correlated in this case with CRP values [6]. Another study used ileocolonoscopy as a comparative investigation and showed the normalization of the ultrasound parameters in 62.8% of the patients, with a significant correlation of the endoscopic modifications [43]. These results also prove the usefulness of the intestinal ultrasound when monitoring the evolution and treatment response in patients with Crohn’s disease.

Transmural inflammatory bowel modifications for this disorder rarely normalize completely despite the immunosuppressive and/or the biological treatment [44–46]. The intestinal ultrasound is an essential imagistic method for assessing transmural healing, which is defined as the reduction of the intestinal parietal thickness <3 mm. Recent studies showed transmural healing in just 25% of the patients and mucous healing in 38%, and the results were not statistically significant [47]. Similar results were also obtained when comparing the intestinal ultrasound with the MRI results. Therefore, transmural and mucous healing was assessed by ultrasound, MRI, and colonoscopy in patients with Crohn’s disease during 2 years of anti-TNF α biological therapy. The study showed a significant correlation between the results of the intestinal ultrasound and the MRI regarding remission of the inflammation, confirming the critical role of the intestinal ultrasound as a non-invasive method that is easy to access and well-tolerated when monitoring Crohn’s disease [45].

A recent study researched the evolution of the subjects with Crohn’s disease with a good response to the anti-TNF α biological treatment and treatment-induced ultrasound changes. The treatment improved the ultrasound in a variable manner: the intestinal wall thickness decreased by 1.5 mm, the Doppler signal normalized in 69.7% of the patients, and the complications were healed in 66.7% of the patients. Nonetheless, the thickness of the intestinal wall decreased in just 42.4% of the subjects, and this showed the lack of correlation between the clinical (the study showed a clinical remission of 87.9%) and the morphologic picture. The transmural healing assessed in ultrasounds was correlated with reduced need for surgery and the intensification of the medical treatment, proving that a normal result of the intestinal ultrasound examination may be translated as a probability of almost 100% of good medium-term therapeutic response [48].

## CONCLUSION

The intestinal ultrasound is a non-invasive method that may be used to monitor patients with Crohn’s disease and may bring important information needed to guide their therapy management. This results from the statistically significant positive correlations of the ultra-sound parameters with standard monitoring parameters of these patients, as well as the established severity scores including clinical, laboratory and endoscopic data and inflammatory markers such as CRP and fecal calprotectin. Moreover, the intestinal ultrasound may assess the entire intestinal wall, unlike the endoscopic techniques by which only the mucosa may be seen and could document transmural healing during treatment.
